# P070: Extended spectrum beta lactamase producing Acinetobacter baumannii in Kuwait

**DOI:** 10.1186/2047-2994-2-S1-P70

**Published:** 2013-06-20

**Authors:** L Vali, AA Dashti

**Affiliations:** 1 Medical Laboratory Sciences, Kuwait University, Kuwait, Kuwait

## Introduction

*Acinetobacter baumannii* is one of the most important opportunistic pathogens causing serious complications. In Kuwait in recent years the prevalence of resistance to antibiotics has raised serious concern especially in the intensive care units. Resistance to carbapenems is mainly caused by the OXA type enzymes; however resistance to cephalosporins are caused by chromosomal AmpC or by extended spectrum beta-lactamases, such as PER.

## Objectives

In this study we investigated the epidemiology of the extended spectrum beta-lactamase PER-like enzymes among the clinical *A. baumannii* recovered in a secondary hospital in Kuwait.

## Methods

One hundred and ten non-duplicate *A. baumannii* isolates were collected from July 2011 to August 2012. Antibiotic susceptibility testing was performed by Vitek2 and examined according to the CLSI guidelines. *gyrB* multiplex PCR was performed to identify *A. baumannii* species. PCR was used to amplify *bla* (OXA-types) carbapenemases, insertion elements, *bla*(NDM), *bla*(PER), *bla*(GES), *bla*(VIM) and *bla*(IMP). PCR products were sequenced and analyzed. Pulsed-field gel electrophoresis (PFGE) was used to genotype the isolates.

## Results

*bla*(OXA-23) was identified in 28 *A.baumannii* isolates, *bla*(OXA-24) in 6, GES-type in 1 and PER-like in 6 isolates. PFGE analysis revealed the strains containing the PER-7 like enzyme which contained OXA-23 belonged to two different PFGE types. Two point mutations on the Ω-loop of the PER-7 protein were detected which can be significant in increasing resistance to cephalosporins.

## Conclusion

We have identified the presence of PER-7 like enzyme in different genotypes of *A.baumannii*. The PER-like genes are located on multi-resistance plasmids and are considered as extended spectrum beta-lactamases causing increased resistance to cephalosporin antibiotics. In Kuwait cephalosporins are generally used to treat *A.baumannii* infections; therefore it is important to monitor and to control the spread of horizontal transfer by administering the correct antibiotic and preventing their spread among hospitalized patients.

## Disclosure of interest

None declared

